# Predicted Anode Arc Attachment by LTE (Local Thermodynamic Equilibrium) and 2-T (Two-Temperature) Arc Models in a Cascaded-Anode DC Plasma Spray Torch

**DOI:** 10.1007/s11666-021-01253-4

**Published:** 2021-09-09

**Authors:** Rodion Zhukovskii, Christophe Chazelas, Vincent Rat, Armelle Vardelle, Ron Molz

**Affiliations:** 1 CNRS, IRCER, UMR 7315, Université de Limoges, 87000 Limoges, France; 2 Oerlikon Metco (US) Inc, Westbury, New York USA

**Keywords:** atmospheric plasma spray (APS), torch modeling, computational fluid dynamics, electric arc model, heat transfer

## Abstract

In DC plasma spray torches, anode erosion is a common concern. It mainly depends on the heat flux brought by the arc and on the dimensions and residence time of the arc attachment to a given location on the anode wall. The latter depend, to a great extent, on the attachment mode of the arc on the anode wall. This paper compares the anode arc attachment modes predicted by an LTE (Local Thermodynamic Equilibrium) and 2-T (two-temperature) arc models that include the electrodes in the computational domain. It deals with a commercial cascaded-anode plasma torch operated at high current (500 A) and low gas flow rate (60 NLPM of argon). It shows that the LTE model predicted a constricted anode arc attachment that moves on the anode ring, while the 2-T model predicted a diffuse and steady arc attachment. The comparison between the predicted and measured arc voltage showed that the 2-T prediction is closer to the actual voltage. Also, the post-mortem observation of a new anode ring of the actual plasma torch operated under the same conditions for a short time confirmed a diffuse arc attachment on a new anode.

## Introduction

Anode erosion is a common concern in plasma spraying. It brings about variation in arc dynamics, voltage and attachment mode on the anode wall. It may also modify the development of the arc column inside the plasma torch and the plasma jet issuing from the torch (Ref 1-4); it finally limits the lifetime of the anode and causes production shutdowns and increased operating cost.

Therefore, different methods are used by the torch manufacturers to reduce the anode erosion (Ref 5, 6). The most common is to limit the residence time of the arc at the same location on the anode wall. An azimuthal displacement of the anode arc attachment is achieved by a swirling injection of the gas. However, the gas swirl tends to progressively decrease along the torch length because of the high viscosity of the hot arc column (Ref 7, 8). Thus, a high swirling component at the gas injection is required in order to have a significant effect on the anode arc attachment further downstream. The arc anode attachment fluctuations can also be promoted by the torch design (self-setting arc length torch design) (Ref 1, 9) and/or the operating parameters (e.g., arc current, nature and flow rates of the plasma-forming gas) (Ref 10) or the use of an external axial magnetic field (Ref 11-16). However, if the axial movement of the anode arc attachment occurs over a large portion of the anode, it affects the stability of the plasma jet and so the injection and processing of the powder or suspension in the plasma jet.

Common ways to limit the arc movement and produce a rather stable plasma jet are either a sudden expansion of the nozzle or an insulating insert between the electrodes (Ref 17). The second method is now common in most of the commercial plasma torches. The nozzle consists, then, in several rings of which the last ring acts as anode.

Actually, the erosion of the anode is mainly controlled by the heat flux brought by the arc attachment, which essentially depends on the arc current, nature of the plasma-forming gas and time of residence of the arc attachment in a specific anode area. The anode erosion also depends on the surface area of the arc attachment at the anode wall and therefore on the attachment mode (diffuse, constricted, etc.). Cascaded-anode plasma torches are generally operated at lower arc current than conventional torches, the plasma enthalpy increase resulting from an increase in arc voltage (typically around 100-120 V as compared with about 70 V for conventional plasma torch) and thus should benefit of a lower anode erosion, the latter being roughly proportional to the square of arc current. Another approach is to split the arc current in several arcs (Ref 5) either by using a multi-cathode or “by dividing the anode ring into three insulated pie-shaped pieces” (Ref 18).

In addition, the nozzle that is traditionally of pure copper because of its high thermal and electrical conductivity, can be protected from erosion by a tungsten liner which has a much higher melting point and heat of fusion than copper (3422 °C and 35.4 kJ·mol^−1^, respectively, vs 1085 °C and 13.05 kJ·mol^−1^ for copper) as it is done in some commercial plasma torches.

Controlling the heat flux to the anode and the way it is dissipated in the electrode cooling system should help to increase the lifetime of the anode. A large body of papers deals with the experimental investigation of the heat flux distribution on the anode wall (e.g., Ref 19-21). Most of the experiments are based on calorimetry methods and yield the total heat flux to anode; they are coupled with other diagnostic methods (e.g., temperature spectroscopic measurement; Thomson scattering measurement; Langmuir probe; miniature heat conduction probe) or other torch configurations (e.g., split anode) to obtain an insight into the different contributions of heat flux to anode. However, such measurements are cumbersome and tricky. Therefore, numerical models stand out as the easiest way to determine the distribution of the heat flux to anode and its various contributions. The key issue of the models is to provide a reliable and accurate prediction of the arc attachment location and area on the anode wall. The mode and dimensions of the arc attachment can be affected by the way the model considers the flow of electric charges from the arc column to the anode wall. Actually, near the anode, the gas temperature is close to the wall temperature because of the intense cooling of the electrode and, thus, the gas electrical conductivity is too low to allow the current continuity. To thwart the gas cooling effect, some tricks have to be used in LTE (Local Thermodynamic Equilibrium) models. They include an artificially high electrical conductivity imposed in the first layer of cells adjacent to the electrode surface (Ref 22-25) and large cells adjacent to the anode wall in order to take into account the ambipolar diffusion and give rise to a high enough temperature and electrical conductivity in these plasma cells (Ref 26, 27). However, such tricks may affect the anode arc attachment, predicted voltage and, plasma temperature and velocity distributions. The limitations of LTE models have driven the development of plasma torch two-temperature (2-T) models (e.g., Ref 28-33). The 2-T models generally assume local chemical equilibrium and consider that the heavy species and electrons can be both described by a Maxwellian distribution but with different temperatures: *T*_*e*_ (electron temperature) and T_h_ (heavy species temperature). If LTE and 2-T models yield similar results in the arc column where the electron density is high (~10^23^ m^−3^) and collisions in plasma numerous enough to thermalize all the species, deviations occur in the zones of high plasma property gradients and/or in the zones where the plasma particle collisions are not sufficient like in the anode zone.

This study aims to compare, for a commercial plasma torch, the anode attachment predicted by the LTE and 2-T arc models that (i) consider the actual 3-D geometry and materials of the torch inside and (ii) couple the electric arc and electrodes.

The second section presents the commercial plasma torch geometry and operating conditions used in this study. The third section describes the numerical model and computational procedure. The fourth section summarizes and discusses the predictions obtained from the LTE and 2-T model for both the arc development and anode attachment. The last section compares the predicted and measured arc voltage and anode attachment mode.

## Plasma Torch Model and Operating Conditions

**Fig. 1:**
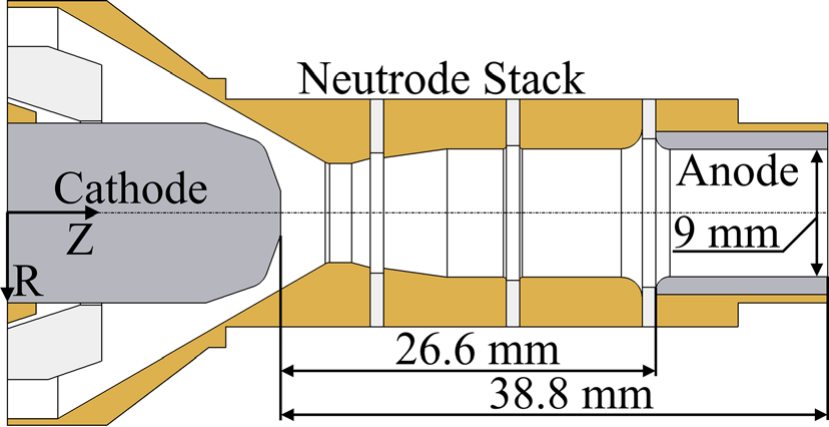
Internal geometry of the SinplexPro™ plasma torch

This work deals with a commercial plasma torch with a single cathode and a cascaded anode (SinplexPro™ plasma torch from Oerlikon Metco) as shown in Fig. 1. The cascaded anode consists of three electrically insulated copper rings, also called neutrodes, and a nozzle which serves as an anode during the torch operation. This anode ring is 9 mm in diameter and 12 mm long. The neutrodes distance the cathode from the anode in such a way as to increase the average arc length and, hence, the plasma enthalpy. The rear neutrode, due to its close position to the cathode, is also used for the torch ignition. During the ignition sequence, a spark is created between the cathode and rear neutrode. The arc length and thus voltage are largely controlled by the neutrode stack between the electrodes and exhibit low fluctuations. The gas pressure in the torch varies also less than in conventional plasma torches.

The cathode of the SinplexPro™ plasma gun is made of tungsten doped with lanthanum(III) oxide. The cathode dopant is used to decrease the material work function (Ref 34) (about 4.5-4.8 eV for pure tungsten compared to 2.72 eV for lanthanum oxide-doped tungsten) and so facilitate the electron emission and improve arcing behavior; it also facilitates the machinability of the electrode material (Ref 35). For this latter reason, the anode liner, which protects the copper anode, is also made of lanthanum oxide-doped tungsten that supports much higher temperatures and heat fluxes than copper. In the SinplexPro™ plasma torch, the plasma-forming gas is injected with an injection angle of 25° through an injector ring that has 24 small orifices.

In this work, the plasma torch was operated with pure argon (60 NLPM) at 500 A. Argon was used as plasma-forming gas due to the two following reasons. First, our previous publications (Ref 16, 36) on the simulation of the operation of the SinplexPro plasma spray torch under LTE assumption dealt with argon, therefore the comparison of the LTE and 2-T models was made for the same plasma gas. Second, the 2-T simulation required the non-equilibrium plasma thermodynamic and transport properties for a small step of the electron temperature and a large array of values of the disequilibrium degree θ between the electron and heavy species temperature (θ = *T*_*e*_/*T*_*h*_), and such properties were readily available in this laboratory.

## Mathematical Model

The mathematical model couples the arc and electrodes (Ref 37). It solves the unsteady Navier–Stokes equations, electric current continuity equation and Ampère's law in both the fluid and electrodes; it is intended to simulate the torch operation with brand-new electrodes with a perfectly smooth surface. The model does not take into account yet the electrode surface erosion, surface deformation due to material melting and crack formation that both strongly affect the arc attachment mode and location and so anode erosion. Therefore, this work aims to simulate the first instants of operation of the plasma torch.

### Main Assumptions

The three-dimensional unsteady fluid model took the actual geometry and materials of the plasma torch inside into account. The properties of the electrode material (electrical conductivity, thermal conductivity, enthalpy and specific heat) were considered as temperature dependent (Ref 38).

The plasma flow was assumed laminar, subsonic and weakly compressible. The plasma was assumed optically thin and in chemical equilibrium, which means that the plasma composition was defined by the plasma temperature and pressure. The plasma composition was assumed to follow the Saha equation with the mass action law approach presented in (Ref 39). The chemical equilibrium can be violated around the electrode arc attachment (Ref 40) and at the arc–electrode interface (Ref 41, 42) due to the strong diffusion of charge carriers. However, the consideration of chemical non-equilibrium in three-dimensional unsteady torch simulations results in significantly higher computational cost. Thus, the present study focused on the comparison of local thermodynamic equilibrium with thermal non-equilibrium predictions, while assuming chemical equilibrium. The electromagnetism phenomena were supposed quasi-steady (Ref 43, 44), i.e., the time derivatives of the electric and magnetic fields were neglected in the electromagnetic equations as generally assumed in arc models, since the dynamics of the model are assumed to be dominated by the thermodynamic configuration of the arc.

In the LTE model, the local thermodynamic equilibrium was assumed in the whole fluid phase of the computational domain. To allow the arc to go through the cold boundary layer and attach at the anode wall, a high electrical conductivity (5000 S/m) was imposed at the very vicinity of the wall while some residual electrical conductivity (195 S/m in this work, which was taken from the 2-T plasma properties for *T*_*e*_=7000 K and *T*_*h*_=1000 K) was supposed to subsist in the anode cold boundary layer behind the anode arc attachment according to the model proposed by Nemchinsky (Ref 45, 46). The value of the artificial electrical conductivity upstream of the anode arc attachment (195 S/m) and the size of the domain where it was imposed were selected from several trials in order to minimize the artificial manipulations with the model, but still ensure stable operation of the model.

### Governing Equations

According to the above assumptions, the governing equations for the fluid model are the following:

#### Mass Conservation Equation

1 $$\underbrace {{\frac{\partial \rho }{{\partial t}}}}_{{{\text{transient}}\,\,{\text{term}}}} + \underbrace {{{\text{div}}\left( {\rho \vec{u}} \right)}}_{{{\text{advection}}}} = 0$$

where $\vec{u}$ is the fluid velocity and ρ the fluid density.

#### Momentum Conservation Equation

2 $$\underbrace {{\frac{\partial }{\partial t}\left( {\rho \vec{u}} \right)}}_{{{\text{transient}}\,\,{\text{term}}}} + \underbrace {{{\text{div}}\left( {\rho \vec{u} \otimes \vec{u}} \right) = - \nabla p}}_{{{\text{advective}}\,\,{\text{terms}}}} + \underbrace {{{\text{div}}\left( {\overline{\overline{{\uptau }}} } \right)}}_{{{\text{viscous}}\,\,{\text{forces}}\,}} + \underbrace {{\left( {{\vec{\Gamma }}_{{{\text{expl}}}} + \overline{\overline{{\Gamma }}}_{{{\text{impl}}}} \vec{u}} \right)}}_{{{\text{user - imposed}}\,\,{\text{source terms}}\,}} + \underbrace {{\vec{j} \wedge \vec{B}}}_{{{\text{Lorentz}}\,\,{\text{force}}}}$$

where $p$ is the pressure; $\overline{\overline{\tau } }$ is the viscous stress tensor: $\overrightarrow{j}$ and $\overrightarrow{B}$ are the electric current density and magnetic field, respectively. The term $\overrightarrow{j}\wedge \overrightarrow{B}$ represents the electromagnetic Lorentz force. The user-imposed source terms $\left( {\overrightarrow {\Gamma }_{{\exp {\text{l}}}} + \overline{\overline{\Gamma }}_{{{\text{impl}}}} \overrightarrow {u} } \right)$ were used to suppress the momentum inside the electrodes according to the penalty method proposed by Patankar (Ref 47) and control the plasma-forming gas injection configuration at the entrance of the computational domain. In case of suppression of the momentum inside the electrodes, the explicit source term ${\overrightarrow{\Gamma }}_{expl}$ imposed in the user subroutine of the CFD code used in this study (Code_Saturne) was a zero vector, while the tensor of the implicit source term $\overline{\overline{\Gamma }}_{{{\text{impl}}}}$ was set to a matrix with − 10^30^ on the main diagonal and zeros in the other positions.

#### Enthalpy Conservation Equation

LTE model: The model assumed that all the species had the same temperature, which was defined as the plasma temperature.

3 $$\underbrace {{\frac{{\partial \left( {\rho h} \right)}}{\partial t}}}_{{{\text{transient}}\,{\text{term}}\,}} + \underbrace {{{\text{div}}\left( {\rho \vec{u}h} \right)}}_{{{\text{advection}}}} = \underbrace {{{\text{div}}\left( {\frac{\lambda }{{C_{p} }}\vec{\nabla }h} \right)}}_{{{\text{thermal}}\,\,{\text{diffusion}}\,}} + \underbrace {{\frac{{\vec{j} \cdot \vec{j}}}{\sigma }}}_{{{\text{Joule}}\,{\text{power}}\,}} + \underbrace {{\frac{5}{2}\frac{{k_{B} }}{\left| e \right|}\frac{{\vec{j} \cdot \vec{\nabla }h}}{{C_{P} }}}}_{{{\text{heat transport}}\,\,{\text{by electric current}}\,}} - \underbrace {{Q_{R} }}_{{{\text{radiation}}}}$$

where $h$ is the plasma enthalpy; $\lambda$, ${C}_{p}$ and $\sigma$ are the plasma thermal conductivity, specific heat and electrical conductivity, respectively; ${k}_{B}$ is the Boltzmann constant and e the elementary charge. ${Q}_{R}$ represents the volumetric net radiation losses calculated from the net emission coefficient.

#### Two-Temperature Model

##### Electron Enthalpy Conservation Equation

4 $$\underbrace {{\frac{\partial }{\partial t}\left( {\rho h_{e} } \right)}}_{{{\text{transitive}}\,\,{\text{term}}\,}} + \underbrace {{div\left( {\rho \vec{u}h_{e} } \right)}}_{{{\text{advection}}}} = \underbrace {{div\left( {\frac{{\lambda_{e} }}{{C_{p}^{e} }}\nabla h_{e} } \right)}}_{{{\text{electron}}\,\,{\text{thermal}}\,\,\,{\text{diffusion}}}} + \underbrace {{\frac{{\vec{j} \cdot \vec{j}}}{\sigma }}}_{{{\text{Joule}}\,\,{\text{power}}}} + \underbrace {{\frac{5}{2}\frac{k}{\left| e \right|}\frac{{\vec{j} \cdot \nabla h_{e} }}{{C_{p}^{e} }}}}_{{{\text{heat}}\,\,{\text{transport\,by}}\,\,{\text{electric current}}}} - \underbrace {{\delta_{e} Q_{R} }}_{{{\text{continuum}}\,{\text{radiation}}\,}} - \underbrace {{K_{{{\text{exchange}}}} \left( {T_{e} - T_{h} } \right)}}_{{\text{exchange\,term}}}$$

where the subscript e refers to electrons and subscript h to heavy species. *λ*_*e*_ is the translational thermal conductivity of electrons.

##### Heavy Species Enthalpy Conservation Equation

5 $$\underbrace {{\frac{\partial }{\partial t}\left( {\rho h_{h} } \right)}}_{{{\text{transitive}}\,\,\,{\text{term}}\,}} + \underbrace {{{\text{div}}\left( {\rho \vec{u}h_{h} } \right)}}_{{{\text{advection}}}} = \underbrace {{{\text{div}}\left( {\frac{{\lambda_{h} + \lambda_{r} }}{{C_{p}^{h} }}\nabla h_{h} } \right)}}_{{{\text{heavy\,species}}\,\,{\text{thermal\,diffusion}}}} - \underbrace {{\left( {1 - \delta_{e} } \right)Q_{R} }}_{{{\text{line\,radiation}}}} + \underbrace {{K_{{{\text{exchange}}}} \left( {T_{e} - T_{h} } \right)}}_{{\text{exchange\,term}}}.$$

$\lambda_{h}$ is the translational thermal conductivity of heavy species and $\lambda_{r}$ the reactive thermal conductivity. The latter was associated with heavy species as suggested by Trelles et al. (Ref 31, 48) and Haidar (Ref 49). $\delta_{e}$ corresponds to the relative share of continuum radiation in the total radiative losses. The radiation losses coming from the lines were attributed to heavy species while the part coming from the continuum was attributed to electrons (Ref 50).

${K}_{exchange}$ is the thermal exchange coefficient between electrons and heavy species in elastic collisions. It was calculated according to the work of Freton et al. for free burning arcs (Ref 51). The ionization energy was associated with electrons as suggested by Freton et al. (Ref 51), who showed it was physically correct and based on the Boltzmann equation. Other works associate the ionization energy to the heavy species (Ref 28, 31, 48) and the question is still open.

Therefore, the enthalpies of electrons ${h}_{e}$ and heavy species ${h}_{h}$ were expressed as follows:

6 $$h_{e} = \frac{5}{2}\frac{{k_{B} }}{\rho }n_{e} T_{e} + \underbrace {{\frac{1}{\rho }\mathop \sum \limits_{\zeta = 0}^{N} n_{h}^{\zeta + } E_{\zeta }^{{{\text{form}}}} }}_{{{\text{ionization}}\,\,{\text{energy}}}}$$

7 $$h_{h} = \frac{5}{2}\frac{{k_{B} }}{\rho }\mathop \sum \limits_{\zeta = 0}^{N} n_{h}^{\zeta + } T_{h}$$

where ${E}_{\zeta }^{form}=\sum_{i=1}^{\zeta }{E}_{i}$ is the formation energy of the species ${Ar}^{\zeta +}$ from the neutral atom ${Ar}^{0}$, the formation energy of the neutral atom being zero. The maximum ion charge considered in this study is +4.

#### Electromagnetic Equations

8 $${\text{Electric\,current\,conservation}}:\,\,\,{\text{div}}\left( {\sigma \nabla \varphi } \right) = 0$$

9 $${\text{Ampere}}^{{\prime}} {\text{s law}}:\,\,\,{\text{div}}\left( {\nabla \vec{A}} \right) = - \mu_{0} \vec{j}$$

10 $${\text{Ohm}}^{{\prime}} {\text{s law}}:\,\,\,\vec{j} = - \sigma \nabla \varphi$$

where ${\mu }_{0}$ is the permeability constant; $\overrightarrow{A}$ is the magnetic vector potential used to derive the magnetic field as $\overrightarrow{B}=\nabla \times \overrightarrow{A}$ (Ref 11) and $\varphi$ is the standard electric potential which is used to derive the electric field as $\overrightarrow{E}=- \nabla \varphi$ (Ref 12).

The non-LTE plasma composition was determined from the Saha equation, Dalton’s law for pressure and electric neutrality condition (Ref 51). The non-LTE plasma transport properties were computed according to the approaches of Bonnefoi (Ref 52) and formulae detailed for example in (Ref 53, 54). The LTE plasma properties were derived from these data for $\theta ={T}_{e}/{T}_{h}=1$. The data for the plasma radiative heat loss were taken from the work of Erraki (Ref 50).

#### Computational Domain and Boundary Conditions

**Fig. 2:**
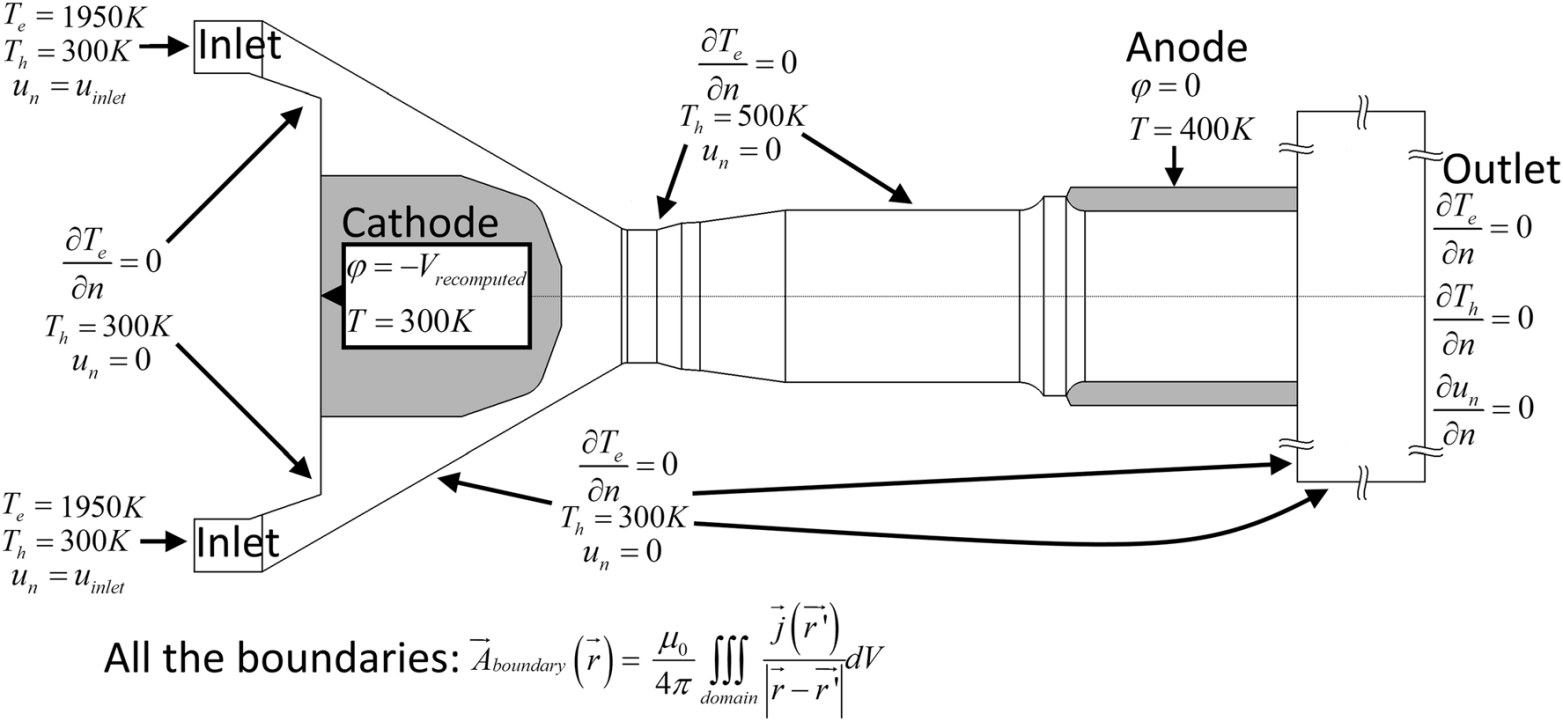
Boundary conditions for the 2-T model

The computational domain and boundary conditions for the set of Eqs. 1, 2, 3, 4, 5, 6, 7, 8, 9 and 10 are shown in Fig. 2. Both the anode and cathode were included in the computational domain that included the arc chamber from the gas injector ring upstream of the cathode to the nozzle exit (58 mm long) and an outside domain (36 mm long). The inclusion of electrodes makes it possible to obtain a better prediction of the magnetic field in the vicinity of the electrodes and electric current density on the electrode surface, and a more accurate prediction of the plasma acceleration by the magnetic pressure (Maecker effect). It also makes it possible to predict the temperature distribution at the electrode surfaces. The computational domain included 2061696 hexahedral cells. The cell size varied from 17 μm at the plasma–cathode interface to 800 µm in the periphery of the outside domain. The mesh was refined until the calculated results became insensitive to the refinement.

#### Flow Velocity Boundary Conditions

The plasma-forming gas entered the computational domain at the left border in the form of 24 small jets with an angle of 25° and exited freely at the right border. At the other borders of the domain, the normal component of the velocity was set to zero with no-slip condition.

#### Temperature Boundary Conditions

They include the conditions at the boundaries of the computational domain and at the electrode-fluid interfaces. The heavy species temperature (gas temperature in the LTE model) was set to a specific temperature at all the boundaries of the domain (Dirichlet boundary condition) except at the outlet where a zero-flux boundary condition (Neumann boundary condition) was adopted. The heavy species temperature of 500 K on the neutrode surface downstream of the cathode tip is an approximate value intended to emphasize that this surface is heated up by the plasma radiation. However, the model was little sensitive to the value of the temperature on the neutrode surface. The temperatures on the external surface of the cathode (300 K) and anode tungsten liner (400 K) are approximate values that intended to mimic the water-cooling system. The temperature on the external anode tungsten liner surface is a little higher because the liner is distanced from the cooling water by a thicker layer of copper compared to the cathode. It should, however, be kept in mind that the approximate values of temperature on the external surfaces of the electrodes may slightly affect the predicted electrode temperatures.

The zero-flux boundary condition was used for the electron temperature at all the boundaries except at the inlet surface where the electron temperature was set to a specific value (~1950 K). This zero-flux boundary condition on the walls of the torch is a simplification. In general, the boundary condition for electron temperature corresponds to some specific value defined by the interaction model between the solid body and plasma. For example, the electron temperature on the cathode surface should be defined by a cathode sheath model (Ref 55); it should be around 10000 K for the electric current density predicted at the cathode tip (Ref 56). The computation of the cathode sheath parameters is beyond the scope of this study. However, the electron temperature adjacent to the cathode surface predicted by the 2-T model should be a little overestimated (around 12500 K in this study). A properly simulated cathode sheath with the calculation of the electron temperature on the cathode surface would result in a lower electron temperature in the vicinity of the cathode and hence in a higher arc voltage. The zero-flux boundary condition for the neutrode stack surface between the cathode and anode can be considered as sufficiently accurate since the thermal balance for electrons near the wall is dominated rather by the Joule power and exchange with heavy species than by enthalpy dissipation and the model was insensitive to the electron temperature on the neutrode surface.

The electron temperature of 1950 K imposed at the gas inlet is meant to indicate the area with the coldest gas. In addition, the electron number per fluid cell below this temperature value is less than one, which makes the concepts of Maxwellian distribution and electron temperature inapplicable. In general, the number density of electrons in the cold distant areas can be artificially increased to achieve the electron temperature equal to the heavy species temperature (Ref 31, 51). However, in this model, such an artificial increase in electron number density does not change the predicted arc parameters while it brings about fluctuations and instabilities in the electron temperature and exchange term in those cold distant areas. An electron temperature of 1950 K at the inlet resulted in the most stable model configuration, while it did not change the predicted parameters of the arc.

#### Electrode–Plasma Interface

At the electrode–plasma interface, the temperature continuity was imposed for the heavy species temperature (plasma temperature in the LTE model) while a zero flux (Neumann boundary condition) was used for the electron temperature.

The anode and cathode sheaths are beyond the scope of this study, thus the cathode and anode voltage drops were assumed constant in time and uniform over the whole surface of each electrode. The electrode heating by electric current was implemented similarly to (Ref 36, 37), except that this model took into account the difference in electron and heavy species temperature and was complemented by the heating of the electrodes due to plasma radiation ${Q}_{r}^{abs}$ and their cooling due to the black-body radiation of the electrodes ${Q}_{bbr}^{em}$. The heat flux to the electrodes at the plasma–electrode interface was expressed as follows:

13 $$Q_{{{\text{cathode}}}} = - J_{{{\text{emis}}}}  \left( {\frac{{2k_{b} }}{e}T_{{{\text{cathode}}}} + \frac{{\Phi_{W} }}{e}} \right) + J_{{{\text{ions}}}} \left( {\frac{{5k_{b} }}{2e}\left( {T_{h} - T_{{{\text{cathode}}}} } \right) + U_{c} + \frac{{\Phi_{i} }}{e}} \right) + Q_{r}^{abs} - Q_{bbr}^{em} + Q_{{{\text{diff}}}}$$

14 $$Q_{{{\text{anode}}}} = J_{{{\text{elec}}}} \left( {\frac{{5k_{b} }}{2e} \left( {T_{e} - T_{{{\text{anode}}}} } \right) + U_{a} + \frac{{\Phi_{W} }}{e}} \right) + Q_{r}^{abs} - Q_{bbr}^{em} + Q_{{{\text{diff}}}}$$

where ${J}_{emis}$ is the thermionic emission current density computed from the Richardson–Dushman law as a function of the computed cathode temperature with the La_2_O_3_ work function ${\Phi }_{W}$=2.72 eV and Richardson constant = 8·10^4^ A m^−2^ K^−2^ (Ref 34). ${J}_{ions}={J}_{calculated}-{J}_{emis}$ is the ion electric current directed to the cathode tip. The secondary electron emission is considered negligible as the predicted current density is generally above 10^6^ A/m^2^. ${U}_{c}$ and ${U}_{a}$ are the cathode and anode sheath voltage drop, respectively. ${\Phi }_{i}$ is the first ionization potential of argon and $e$ is the electron charge.

In addition, the electrodes were heated up by the thermal diffusion from the plasma heavy species to the electrode. The heat flux from the plasma to electrode due to thermal diffusion was expressed as:

15 $$Q_{{{\text{diff}}}} = \frac{{2\lambda_{{{\text{elec}}}} \lambda_{hr}^{pl} }}{{\lambda_{{{\text{elec}}}} l_{pl} + \lambda_{h}^{pl} l_{{{\text{elec}}}} }}\left( {T_{plh} - T_{{{\text{elec}}}} } \right)$$

where ${\lambda }_{elec}$ is the thermal conductivity of the electrode interface cell, ${\lambda }_{hr}^{pl}$ the sum of reactive and heavy species translational thermal conductivities in the plasma interface cell, ${T}_{elec}$ the temperature in the electrode interface cell, ${T}_{plh}$ the heavy species temperature in the plasma interface cell, ${l}_{pl}$ and ${l}_{elec}$, respectively, the plasma and electrode interface cell sizes perpendicular to the interface surface.

The continuous transition of the plasma heavy species temperature to the electrode temperature in this case is a simplification, since it neglects the anode and cathode sheaths. Thus, the thermal balance at the electrode surface is rather approximate and should require further development.

Since the cathode sheath was not considered in this study, the cathode sheath voltage drop was just added to the simulated voltage in the post-processing.

#### Electromagnetic Boundary Conditions

This model used a procedure that dynamically recalculates the voltage imposed on the outside surfaces of the electrodes in order to maintain a given value of the electric current intensity. The procedure consisted in calculating the integral of the Joule effect in the whole simulation domain and comparing it to the product of the prescribed current intensity and voltage applied to the electrodes. The value of the arc voltage was then decreased if the total Joule power was higher than this product and increased otherwise. A detailed description of this procedure is given in (Ref 36, 47, 57). At the plasma–electrode interfaces, the continuity of electric current was applied.

The magnetic vector potential was dynamically calculated by the Biot–Savart law at all the boundaries of the domain according to the following equation (Ref 36, 58).

16 $$\vec{A}_{{{\text{boundary}}}} \left( {\vec{r},t} \right) = \frac{{\mu_{0} }}{4\pi }\iiint_{{{\text{domain}}}} {\frac{{\vec{j}\left( {\overrightarrow {{r^{\prime}}} ,t} \right)}}{{\left| {\vec{r} - \overrightarrow {{r^{\prime}}} } \right|}}}{\text{d}}V.$$

The set of fluid and electromagnetic equations with the above boundary conditions was solved with the free open-source software Code_Saturne (Ref 57). It is developed and released by EDF and includes various computational fluid dynamics (CFD) applications. It is based on a co-located Finite Volume approach that handles meshes with any type of cell and any type of grid structure and uses a theta scheme for time discretization (finite difference discretization of the time derivative).

The calculations were performed with a dual 22-cores, 44-thread Intel^®^ Xeon^®^ Gold 6152 Processor and 192 GB of RAM. It took around 27 hours per 10000 time steps with a time step of 10^−7^ s.

## Results and Discussion

The main objective of this study is to compare the location and geometry of the anode arc attachment predicted either by the LTE or 2-T temperature model as they both greatly affect the anode erosion between the restarts of the plasma torch. This section first compares the electromagnetic fields in the electrodes and fluid phase and radial profiles of physical properties on the cathode tip surface calculated by the LTE and 2-T models. It also presents the predicted arc current density at the wall of the anode ring, which ends the stack of copper rings insulated from each other, that forms the plasma torch nozzle. Then, the actual arc voltage and pictures of a new anode ring operated with exactly the same conditions as that of the model (500 A; 60 NLPM of argon) are used as a first attempt to validate the predictions. Finally, this section ends with a comparison of the LTE and 2-T temperature and velocity fields in the plasma torch and at nozzle exit.

### Electric Current Density Streamlines and Self-Induced Magnetic Fields

**Fig. 3:**
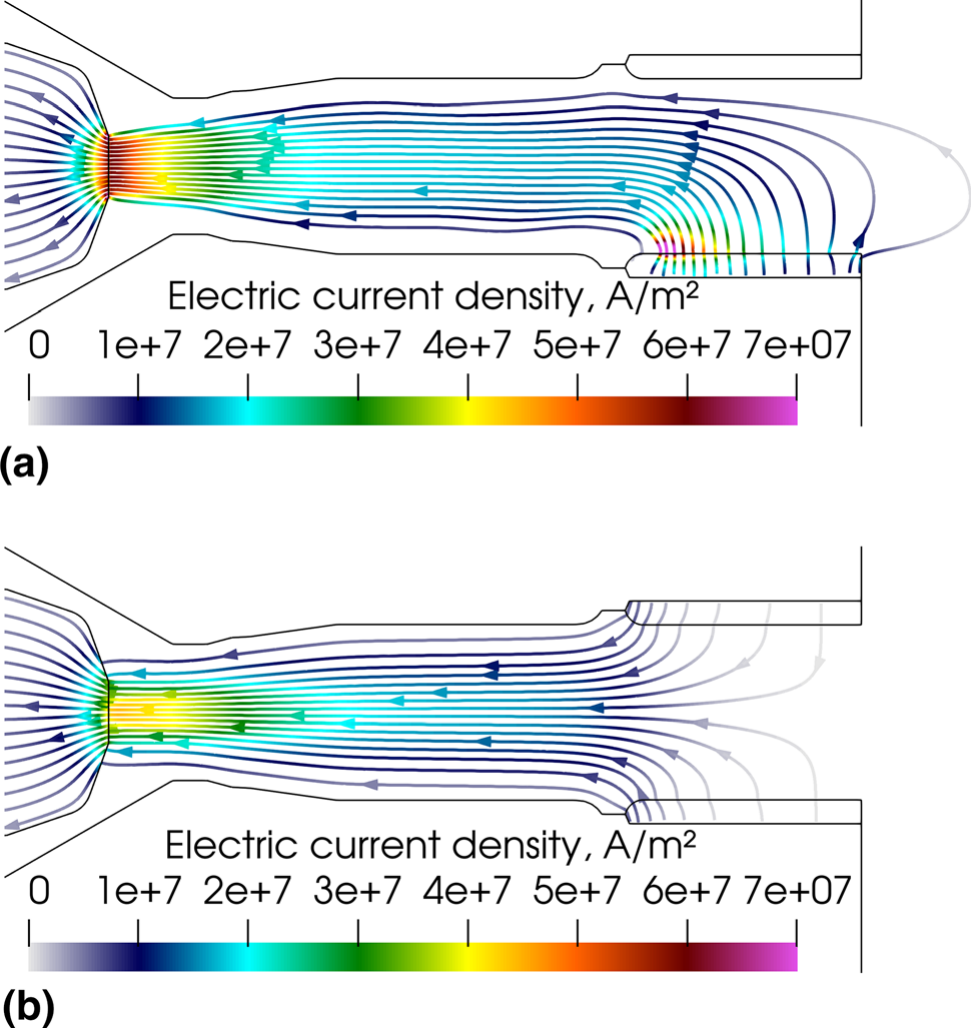
Instantaneous electric current density streamlines in the gas phase and electrodes, 5.2 ms after the arc ignition. (a) LTE model and (b) 2-T model

Figure 3 shows the instantaneous electric current density streamlines, both in the electrodes and fluid phase, calculated with the LTE (Fig. 3a) and 2-T (Fig. 3b) models, respectively. The curvature of the electric current streamlines near the cathode tip is well predicted by both models that also both project a straight arc column in the torch channel. The arc fills most of the cavity in the inter-electrode insert (neutrodes).

However, marked differences can be observed between the two pictures:

- The LTE arc is clearly three-dimensional while the 2-T arc is axisymmetric;
- The LTE anode arc attachment is constricted while the 2-T anode arc attachment is diffuse;
- In the LTE model, the arc attaches to the inner wall of the anode ring and partially fills the anode cavity while in the 2-T model, it attaches all around the upstream edge of the anode ring and hardly penetrates the anode cavity;
- Also, the 2-T cathode arc attachment is slightly wider than the LTE cathode arc attachment and the arc is slightly wider in the 2-T model. In this model, the thermal diffusion of electrons, which have a higher thermal conductivity than heavy species above 15000 K, brings about a heat gain and so a higher electrical conductivity in the arc fringes resulting in the widening of the cathode arc attachment.

**Fig. 4:**
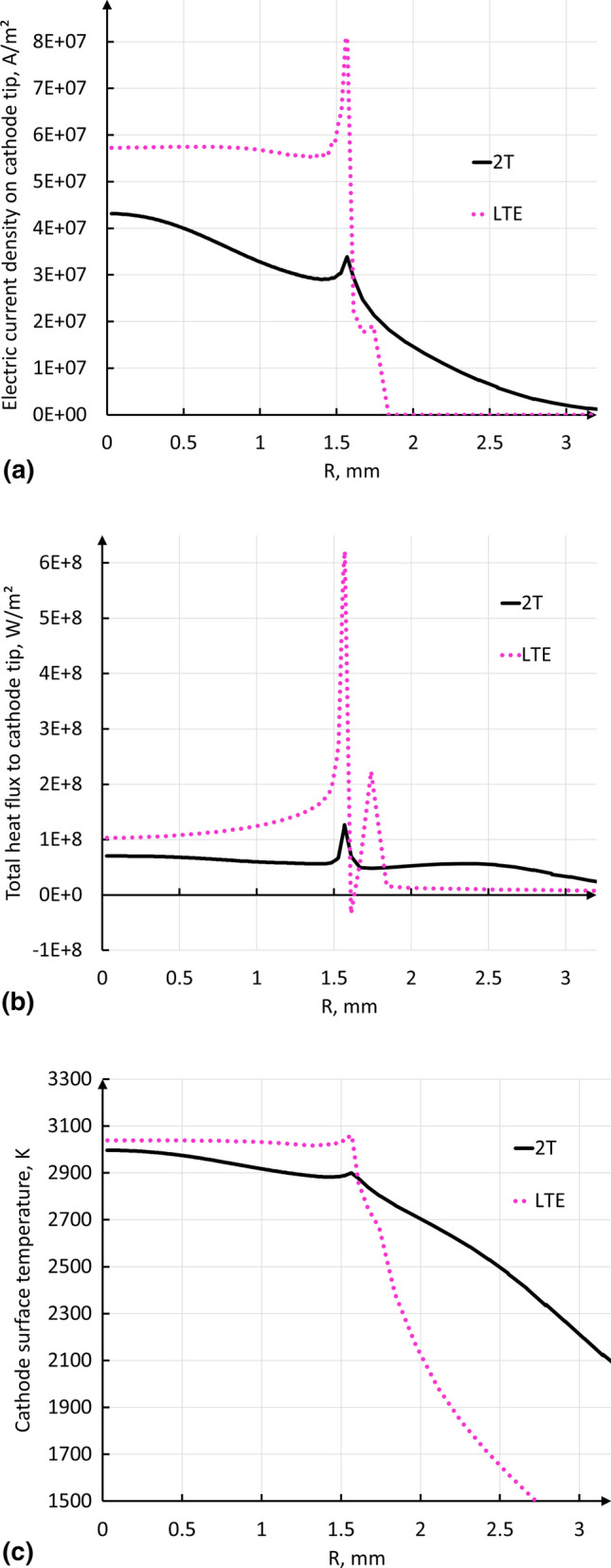
Radial profiles at the interface between the cathode tip and plasma predicted by the LTE and 2-T models (500 A; 60 NLPM of argon). (a) Electric current density (A/m^2^). (b) Total heat flux (W/m^2^). (c) Cathode surface temperature (K)

**Fig. 5:**
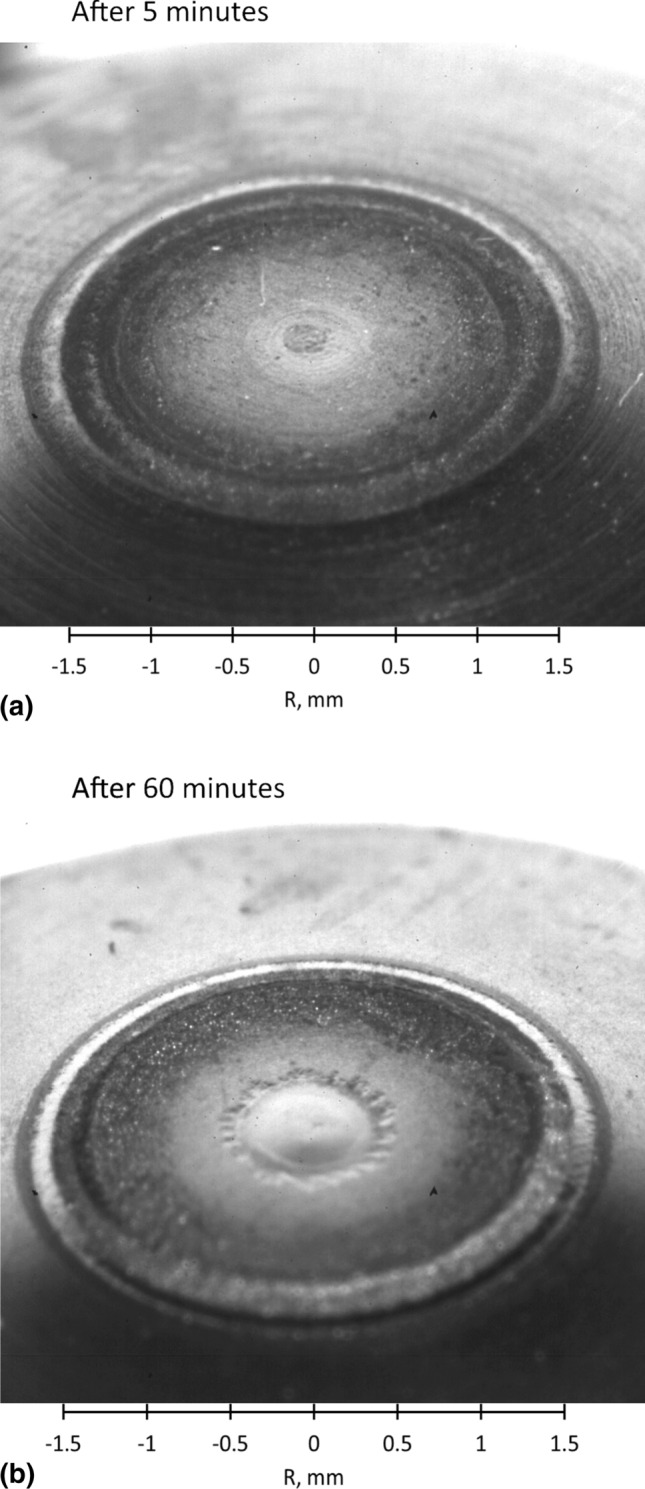
Photographs of SinplexPro cathodes tested for 5 and 60 min with 500 A and 60 NLPM of argon

Figure 4 shows the radial profiles of the electric current density (Fig. 4a), total heat flux to the cathode (Fig. 4b) and cathode surface temperature (Fig. 4c) at the cathode tip calculated by the LTE and 2-T models. The total heat flux to cathode involves the heat flux brought by the electric current, heat flux due to thermal diffusion and radiation. Both models predict a peak in the current density as well as in the heat flux and temperature profiles at the edge of the flat part of the cathode tip (see Fig. 1). However, the radial profile of electric current density calculated by the 2-T model is wider, with a lower current density at the cathode center (4.3×10^7^ A/m^2^ instead of 5.8×10^7^ A/m^2^ in the LTE model). It also has a much lower peak at the edge of the flat part of the cathode tip (3.4×10^7^ A/m^2^ instead of 8.0×10^7^ A/m^2^ in the LTE model). In the 2-T model, the rate of heat transfer to the cathode tip is 1.9 kW (1.1 kW by the electric current, 0.6 kW by heat diffusion and 0.2 kW by radiation) while it is 1.7 kW in the LTE model (1.2 kW by the electric current, 0.1 kW by heat diffusion and 0.4 kW by radiation). The pattern of a hot center with a hot ring observed on the actual cathode tips operated with the same parameters (Fig. 5) is predicted by both models. Nevertheless, the lower peak in the profile of the 2-T heat flux to the cathode makes the high temperature ring at the cathode tip less pronounced contrary to the predictions of the LTE model.

Figure 5 shows the tip of cathodes tested for 5 and 60 min under the conditions of this study (500 A and 60 NLPM of pure argon). The traces of heat load are visible: a small spot at the center of the cathode tip and a ring between the flat and conic parts of the cathode tip. These heating traces match well with the radial profiles of electric current density and total heat flux on the cathode tip surface predicted by the 2-T model. In these profiles, the elevated center and a peak at the edge of the flat part of the cathode tip are clearly visible. The traces on the cathode tip after 5 and 60 min seem to be a result of tungsten recrystallization, which could also facilitate creep deformation of tungsten. The maximum cathode temperatures predicted in both LTE and 2-T models are around 3000 K, which is high enough for tungsten recrystallization. In the photograph of the cathode tested for 5 min, no significant deformation is visible. Thus, even if the cathode melting took place during the 5-min test, it was confined to the immediate surface only. Meanwhile, the cathode tested for 60 min exhibited a small crater with a smooth center. The smooth center of the crater could be created by surface melting, which could be caused by localized depletion of dopant at the surface after 60 min of operation. The depletion of the dopant in the cathode was not considered in the model. Therefore, such surface melting could not be predicted. The jagged crater rim resembled the result of a recrystallization process and, so, that area never reached melting, even at the surface.

**Fig. 6:**
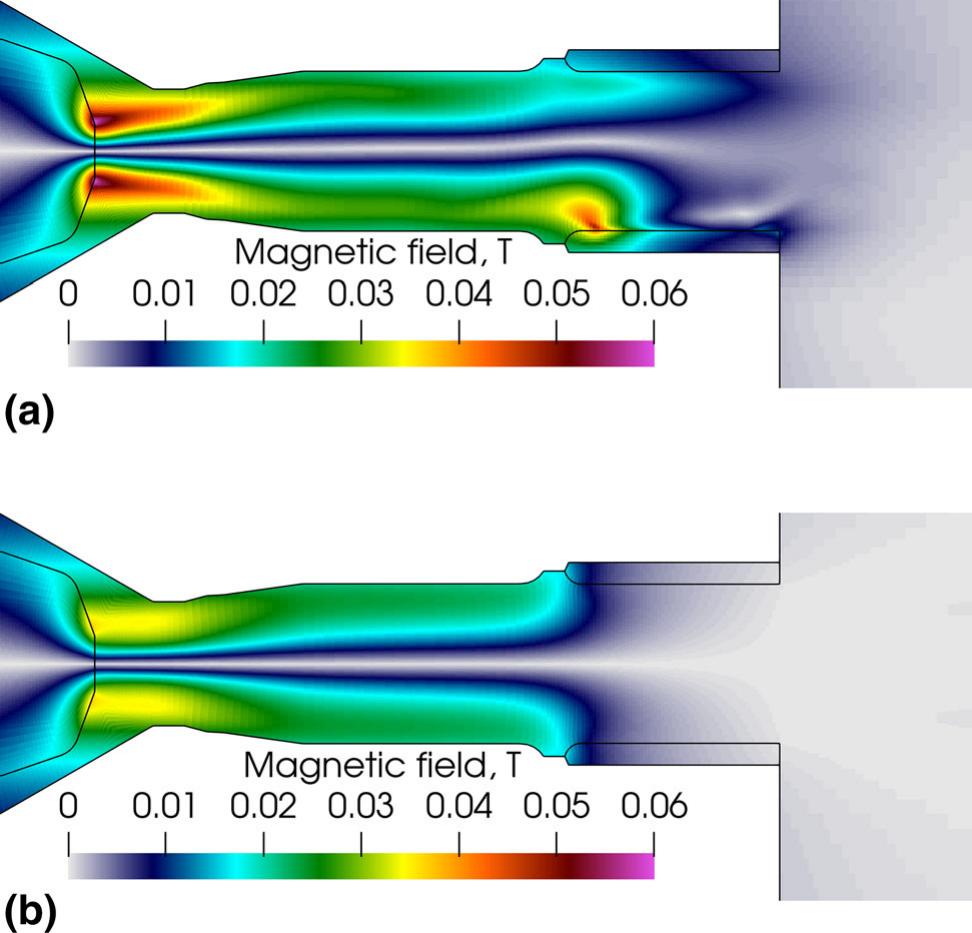
Instantaneous self-induced magnetic field in the gas phase and electrodes, 5.2 ms after the arc ignition. (a) LTE model and (b) 2-T model

Figure 6 shows the self-induced magnetic field in the electrodes and fluid phase for the LTE (Fig. 6a) and 2-T model (Fig. 6b). The magnetic field within the inter-electrode insert shows resemblance with a profile similar to that predicted for a cylindrical conductor with uniform current density. The radial variation has a zero value at the center of the discharge, a radial increase in the arc column with a maximum on the edges of the column and finally a decrease from the periphery of the arc column to the inter-electrode insert wall. The maximum values in both models are found near the tip of the cathode due to the Maecker effect (Ref 59). They reach 0.04 T in the 2-T model and 0.06 T in the LTE model close to the cathode tip but also, 0.05T upstream of the constricted anode arc attachment in the LTE model where it generates the anode jet. In addition, in the 2-T model, the magnetic field is axisymmetric and extends from the cathode to the upstream edge of the anode ring where the electric arc attaches.

In both models, the magnetic field due to the current circulation in the cathode exhibit high values at the edge of the flat part of the cathode. Therefore, near the cathode tip, it contributes to the plasma acceleration that is mainly produced by the interaction of the self-induced magnetic field and electric current density (Lorentz force).

### Anode Arc Attachment Mode

The striking difference between the LTE and 2-T models is a different type of arc attachment on the anode ring. The arc presents a single and constricted attachment in the LTE model while it has a diffuse attachment all around the upstream edge of the anode in the 2-T model.

**Fig. 7:**
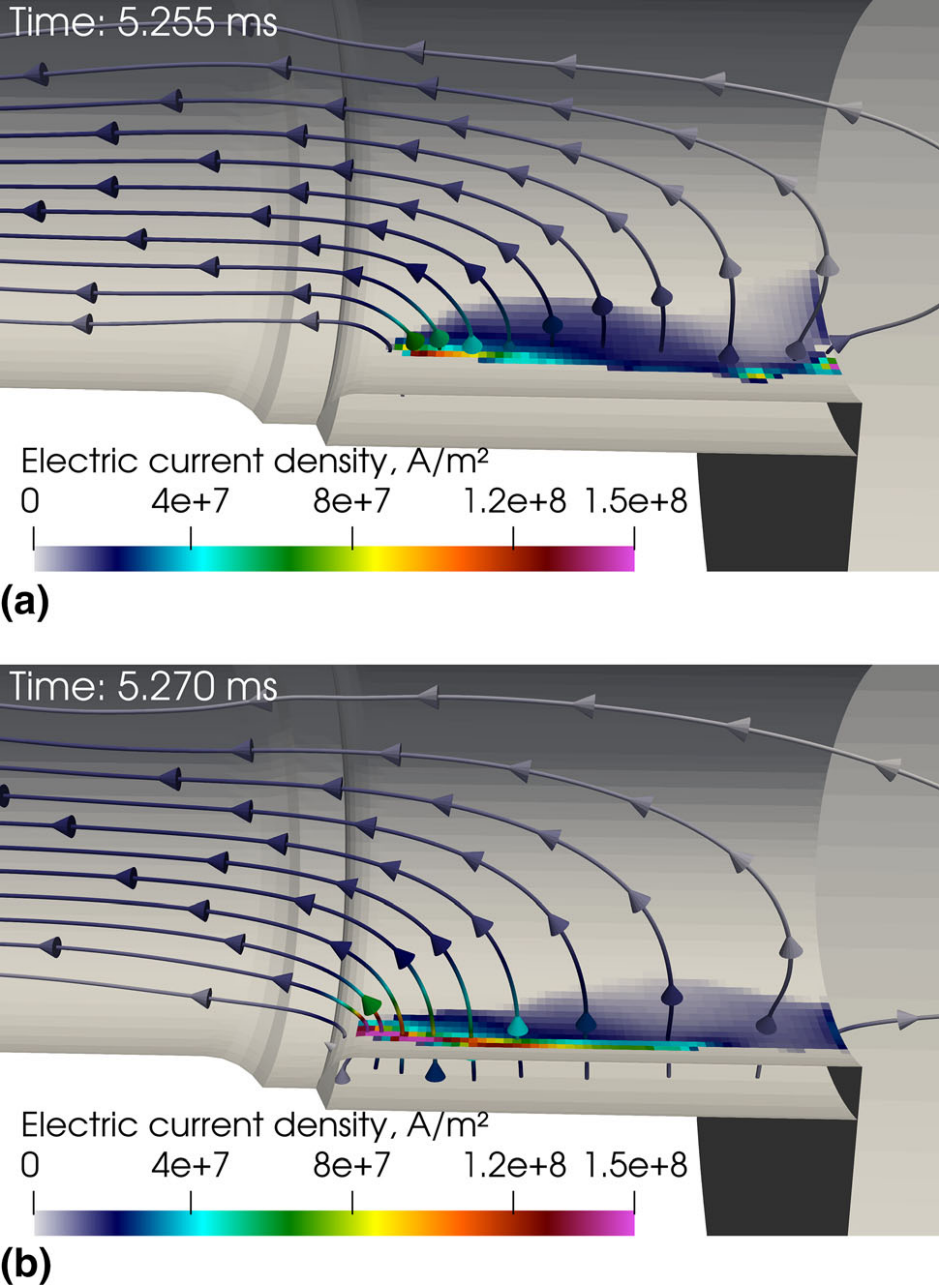
Instantaneous electric current density streamlines and distribution of electric current density on the anode surface in the LTE model at two different instants

Figure 7(a) and (b) shows the LTE current streamlines in the plasma near the anode and the current density field in the anode wall at two different instants. The maximum electric current density at the anode arc attachment is about 1.5 × 10^8^ A/m^2^. The arc attachment exhibits a back and forth movement on the anode ring combined with a rotational movement due to the swirling flow action. Indeed, in this work, the plasma-forming gas is injected with an angle of 25° through 24 holes. The swirling number at the gas inlet, defined as the ratio of the axial flux of the tangential momentum to the axial flux of the axial momentum normalized by the anode radius (Ref 16), is as high as 4.16 in the LTE model. It imparts a strong swirling motion to the cold gas that is progressively dampened by the gas fast acceleration and high viscosity; the swirl number falls at 0.15 in the middle of the channel and 0.06 at the anode ring but is still high enough to make the arc rotate on the anode ring wall.

**Fig. 8:**
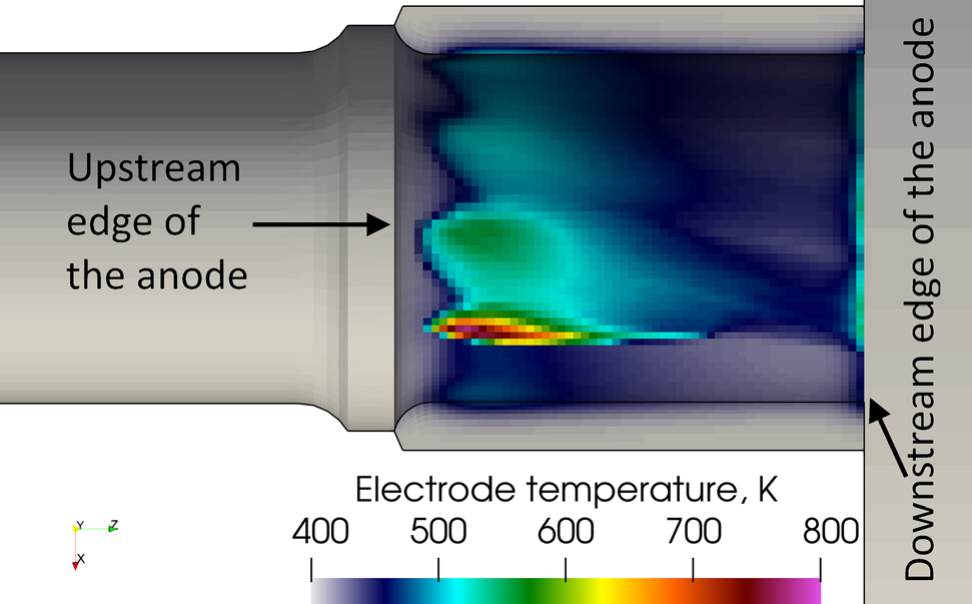
Instantaneous temperature distribution on the anode surface in the LTE model, 5.2 ms after the arc ignition

The localized arc attachment results in a fast heating of the area where it attaches thanks to the rate of heat transfer due to the electric current (3.71 kW) and the rate of heat transfer due to thermal diffusion (3.04 kW). The constriction of the arc in the vicinity of the anode results in a local increase in the Joule effect and so in plasma temperature. However, due to the anode arc attachment rotation resulting from the swirling gas injection the maximum anode temperature simulated in the LTE model (Fig. 8) is far from the tungsten melting point.

In this LTE model, an artificially high electrical conductivity (5000 S/m) was imposed in the first layer of cells adjacent to the anode surface (see the assumption section). This trick is generally used in LTE arc models to allow the connection of the arc column to the anode despite the low temperature and, thus, too low electrical conductivity of the gas in the anode boundary layer (e.g., Ref 22–25). In addition, an artificial electrical conductivity (195 S/m) was also imposed in a small domain upstream of the anode arc attachment. It allows the flow to gain some heat from Joule heating prior to interacting with the anode arc attachment. This makes it possible to mimic the disequilibrium effect behind the anode arc attachment (Ref 45). In this LTE model of the SinplexPro™, without such anode numerical treatment, the arc attaches to the anode outside of the torch and cannot come back inside because the gas boundary layer is too cold to conduct any electric current. The combination of both tricks yields the constricted anode arc attachment in the LTE model, while in the 2-T model, that does not require any assumption on the plasma electrical conductivity close to the electrodes, the arc attachment is diffuse and mostly located at the upstream edge of the anode ring.

**Fig. 9:**
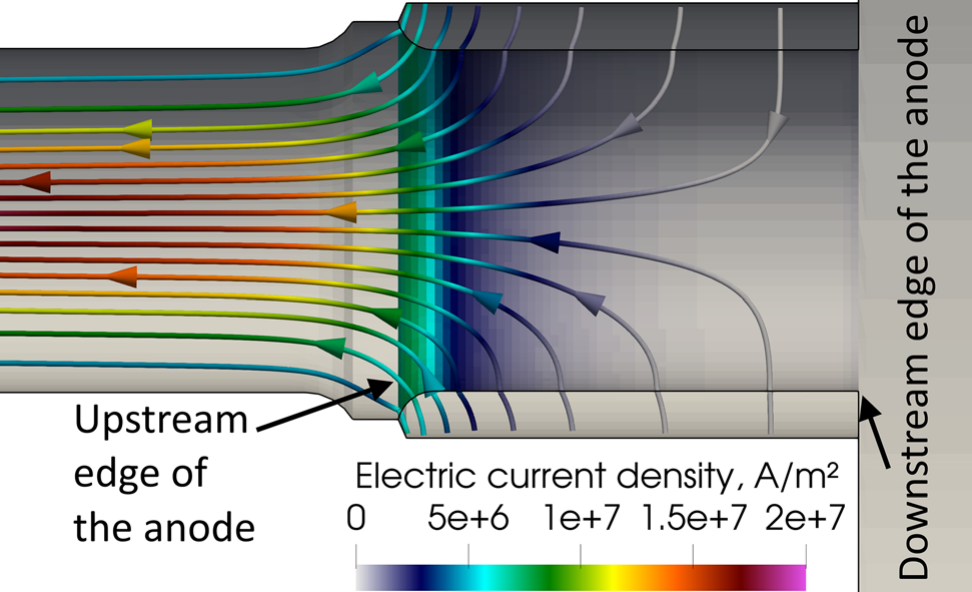
Instantaneous electric current density streamlines and distribution of electric current density on the anode surface in the 2-T model, 5.2 ms after the ignition of the arc

Figure 9 shows the 2-T instantaneous electric current density streamlines and distribution of electric current density on the anode surface. The maximum electric current density is about 8×10^6^ A/m^2^. This value agrees with the experimental data for diffuse arc attachments in argon electric arcs given by Neumann (Ref 60) and Yang et al. (Ref 61).

The attachment mode depends on the boundary layer thickness (e.g., Ref 20). The conditions used for the plasma torch combine a high arc current (500 A) and a rather low gas flow rate (60 NLPM) and should result in a rather thin boundary layer which favors the diffuse arc attachment mode.

**Fig. 10:**
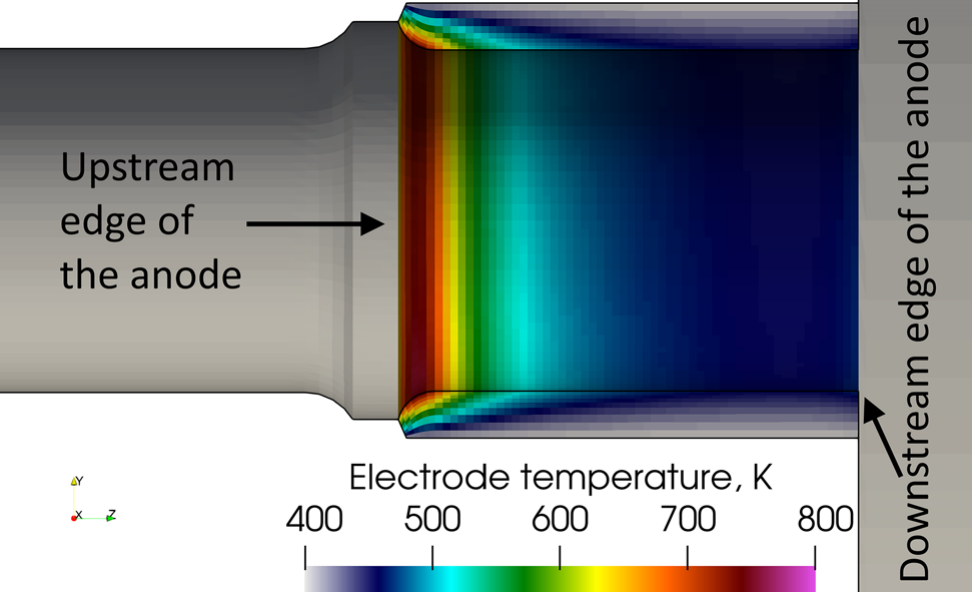
Instantaneous temperature distribution on the anode surface in the 2-T model, 5.2 ms after the ignition of the arc

The temperature distribution on the anode surface in the 2-T model is shown in Fig. 10. The maximum temperature is about 800 K, much too low to cause the melting of the tungsten liner. It is worth noting that, the maximum predicted anode temperature is affected by the temperature boundary condition imposed on the external surface of the anode. Since the boundary condition is assumed at 400 K, the predicted temperature drop over the tungsten liner body is around 400 K. The predicted temperature drop could be employed in other situations with a higher temperature on the external surface of the tungsten liner. The rate of heat transfer due to the electric current is about 2.34 kW and that due to thermal diffusion 1.12 kW. They are both lower than the corresponding rate of heat transfer calculated in the LTE model mainly because of the lower electric current density and, thus, lower temperatures in the vicinity of the anode. Another difference with the LTE model is that the 2-T arc is quasi-steady. The swirling number that is 4.5 at the gas inlet decreases to 0.058 in the middle of the channel and to 0.028 at nozzle exit, which is two times lower than in the LTE model. The lower swirl numbers in the 2-T model can be explained by thermal non-equilibrium effects in the cold boundary layer due to the insufficient collisions between the electrons and heavy species (Ref 62), which yields a higher electron temperature, higher electrical conductivity, higher Joule power and more intense axial acceleration of the cold boundary layer than in the LTE model. The higher axial momentum resulted in lower swirl numbers.

## Validation of Predictions Against Experimental Observations

**Fig. 11:**
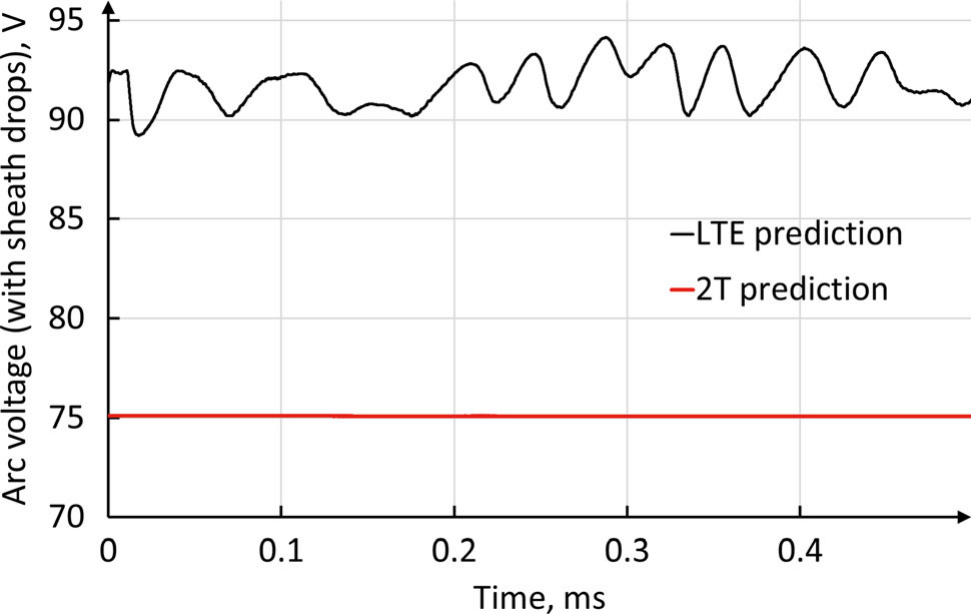
Predicted arc voltage by the LTE and 2-T model

Figure 11 shows the time-evolution of the arc voltage predicted by the LTE and 2-T models. The calculated voltage takes into account the voltage drop at the cathode and anode; they were assumed to be 10 V at the cathode (Ref 56, 60, 63) and 3 V or 0 V at the anode in the LTE or 2-T model, respectively (Ref 60, 64). The values of the cathode voltage drop given in the literature are usually presented as a function of the electric current density. For the current density predicted in this study, they range from about 8 to 10 V. A proper computation of the cathode sheath parameters including their distributions on the cathode surface would improve the predictions. The assumed anode voltage drop was different in the LTE and 2-T model because of the significant differences in the temperature gradient, electric current density and anode arc attachment mode (Ref 65). In the LTE model, the arc motion yielded fluctuations of small amplitude (4%) in the time-evolution of arc voltage, while the arc voltage was constant in the 2-T model. The time-averaged predicted arc voltage was about 93 V and 75 V in the LTE and 2-T model, respectively, while the mean measured torch voltage under the same operating conditions was about 71 V. The higher arc voltage in the LTE model can be explained by a higher resistance to the electric current flow compared with the 2-T model because of the narrower cross section of the arc (corresponding to the region with high electron temperatures) and lower electrical conductivity in the cold boundary layer as the electrons are assumed to have the same temperature as heavy species. In addition, the total radiation heat loss inside the torch was around two times higher in the LTE model (15.7 kW) than in the 2-T model (7.5 kW).

**Fig. 12:**
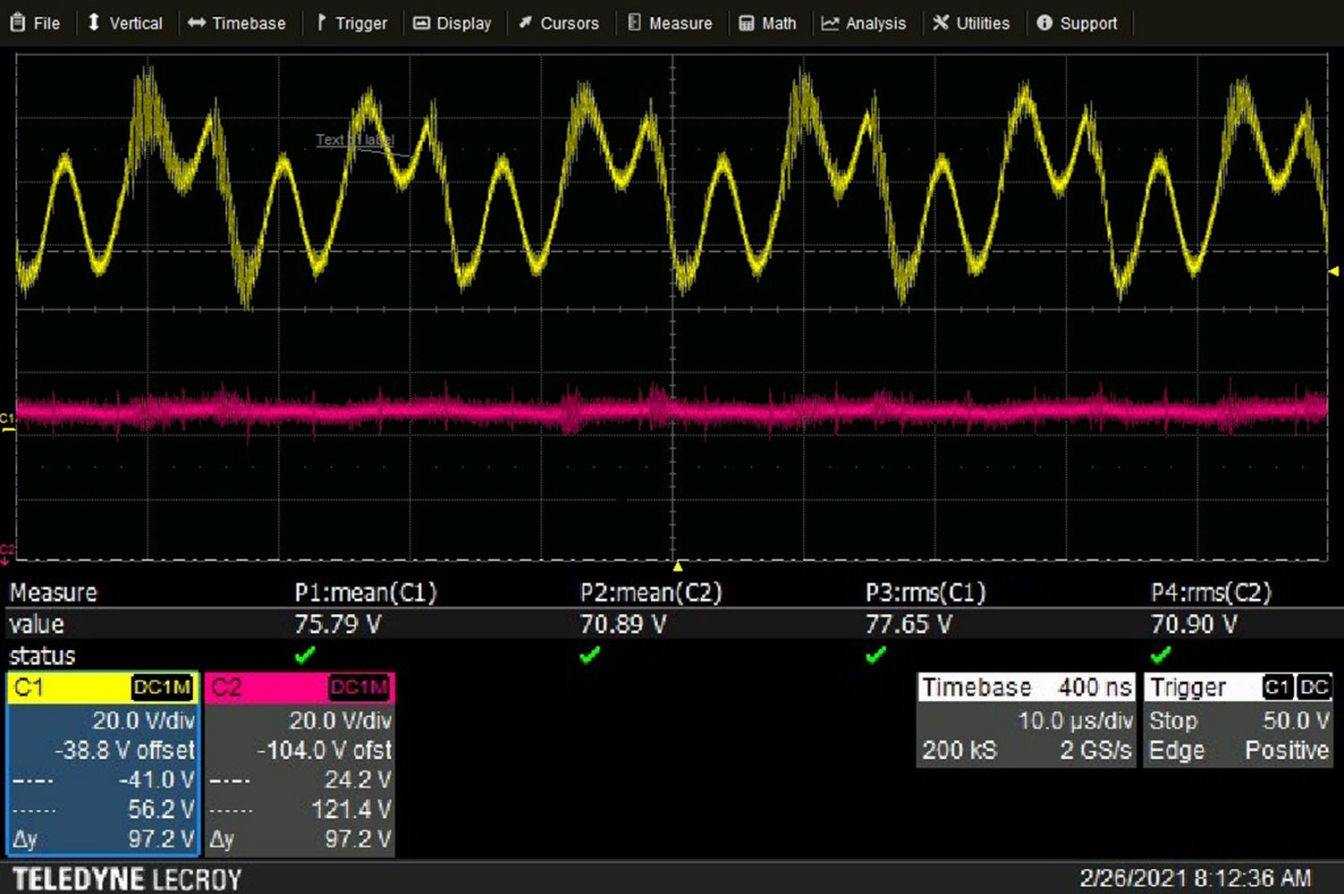
Actual arc voltage. 500 A and 60 NLPM of argon. Yellow curve is voltage measured at the monitoring unit (JAMbox) of the plasma spray system. Pink trace is voltage drop measured directly between the plasma torch electrodes without power supply ripple. Courtesy of Oerlikon Metco

**Fig. 13:**
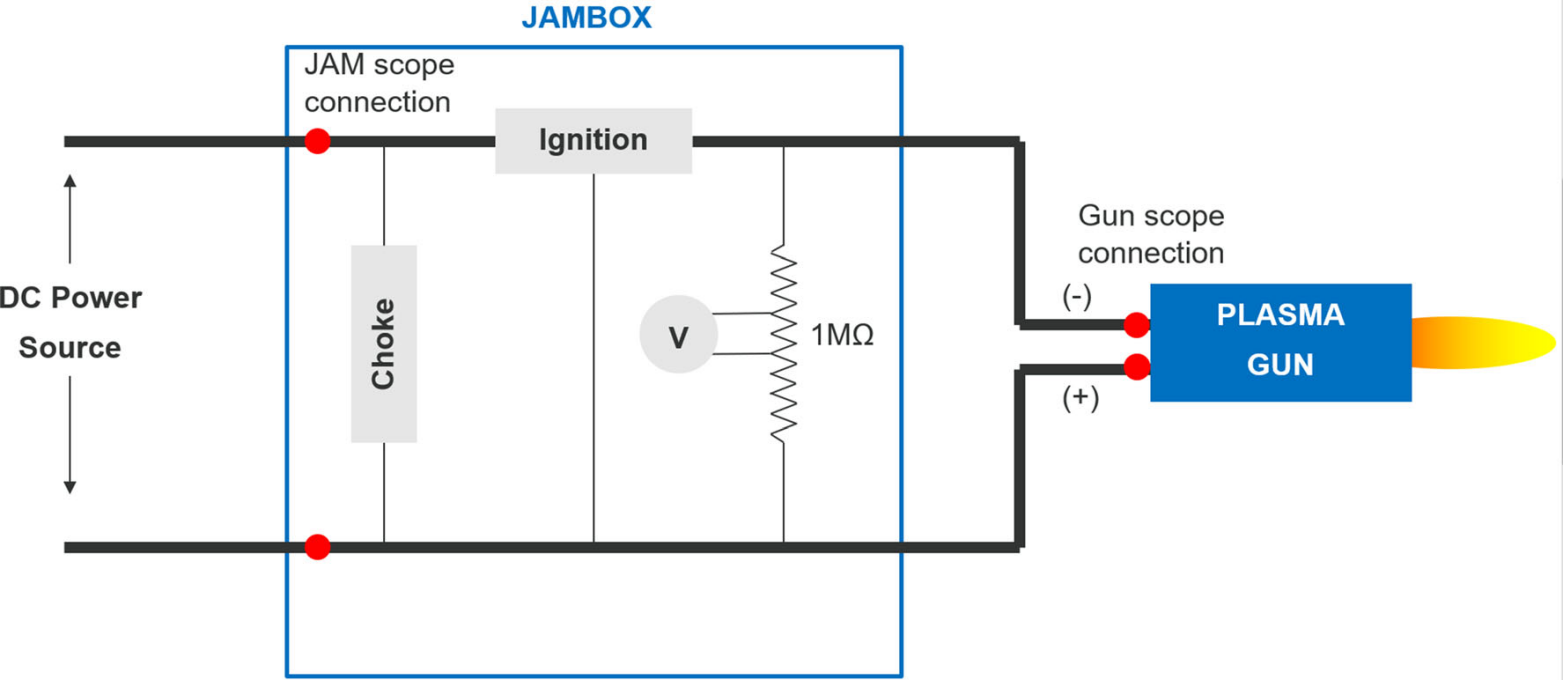
Diagram of electrical circuit used for voltage measurements directly on the torch. JAM stands for Junction and Monitoring. Choke includes a capacitance bridge and a coil and intended to filter out the ripple of the power source

Figure 12 shows the traces from the oscilloscope measuring the plasma torch voltage. The yellow trace corresponds to the voltage measured at the monitoring unit of the plasma spray system; it includes the voltage drop of both the torch and power supply cables. The oscillations are due to the power supply ripple. The pink trace shows the voltage drop measured directly between the plasma torch electrodes, without the power supply ripple and voltage drops of the cables. The actual plasma torch voltage is rather smooth in comparison with the voltage of the whole system. The power supply ripple was minimized by a choke which includes a capacitance bridge and coil designed to block high-frequency pulses. The diagram of the voltage measurements is shown in Fig. 13. The root mean square of the whole plasma spray system was 77.65 V and the mean value 75.79 V while the root mean square of the actual torch voltage was 70.90 V and mean value 70.89 V.

Even if the 2-T model predicted a torch voltage closer to the actual one and a time-evolution similar to the actual one, the difference between the predicted and measured torch voltages was about 4V. As explained by Trelles et al. (Ref 31), this difference could result from the model of the plasma–electrode interfaces and metal vapor issuing from the evaporating dopant (lanthanum oxide) near the electrodes that yields an increase in the plasma electrical conductivity. The inaccuracy of the assumed cathode and anode voltage drops could also contribute to the difference between the calculated and measured voltage.

**Fig. 14:**
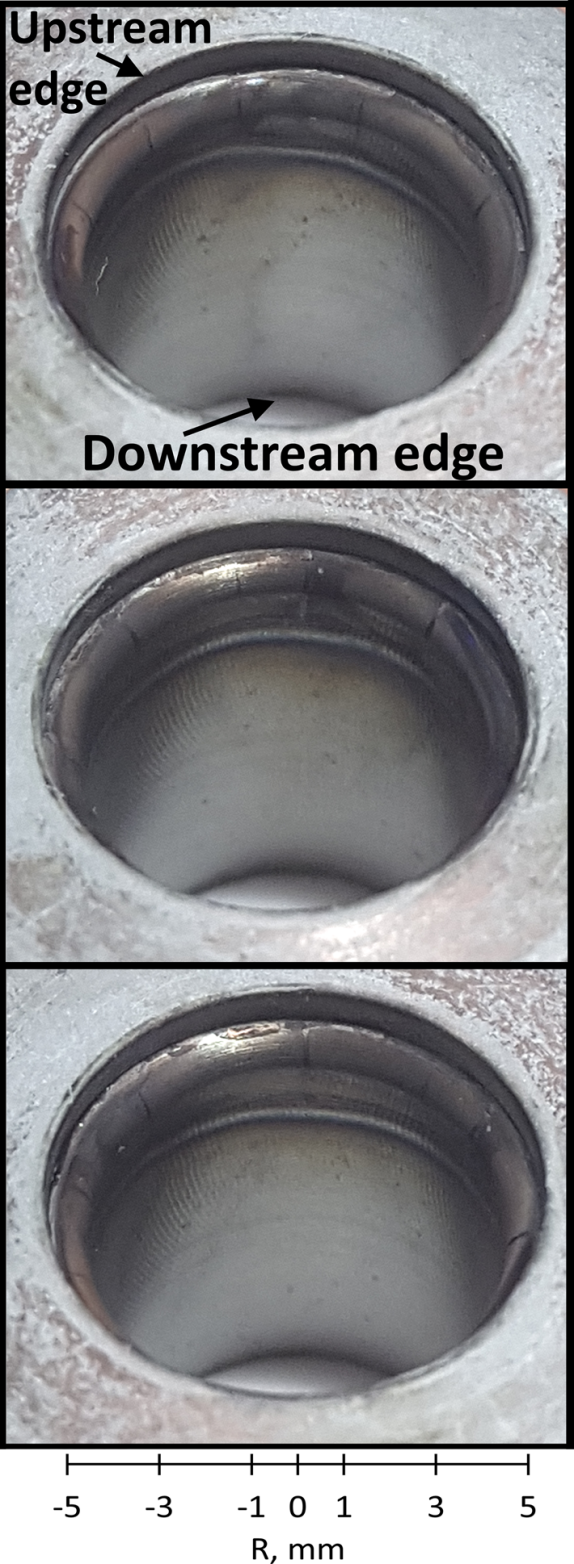
Photographs of SinplexPro new anodes tested for 5 min with 500 A and 60 NLPM of argon from three different angles. The whole circumference is shown

Figure 14 shows three views from different angles of the upstream edge of a new nozzle operated for five min under the same conditions than the ones used in this study. The predicted anode arc attachment in the 2-T model (Fig. 9) resembles the heating pattern on the tested anode: the heating trace is found along the whole circumference of the upstream edge of the anode. No mark of melting appears on the new anode after five min of operation. This agrees with a predicted maximum anode temperature below the tungsten melting point.

Some linear cracks in the upper part of the anode liner are also visible after the cooling down of the anode. These cracks are commonly observed in tungsten-lined plasma nozzles after use. They could be explained by the difference in the thermal expansion of tungsten and copper (about 17×10^−6^ K^−1^ for copper against 4.5×10^−6^ K^−1^ for tungsten) and/or the thermal stresses induced by the colder lower part of the tungsten liner. The cracks may later affect the anode arc attachment: the arc is then attracted by the cracks and exhibits a more constricted mode.

### Temperature Fields

**Fig. 15:**
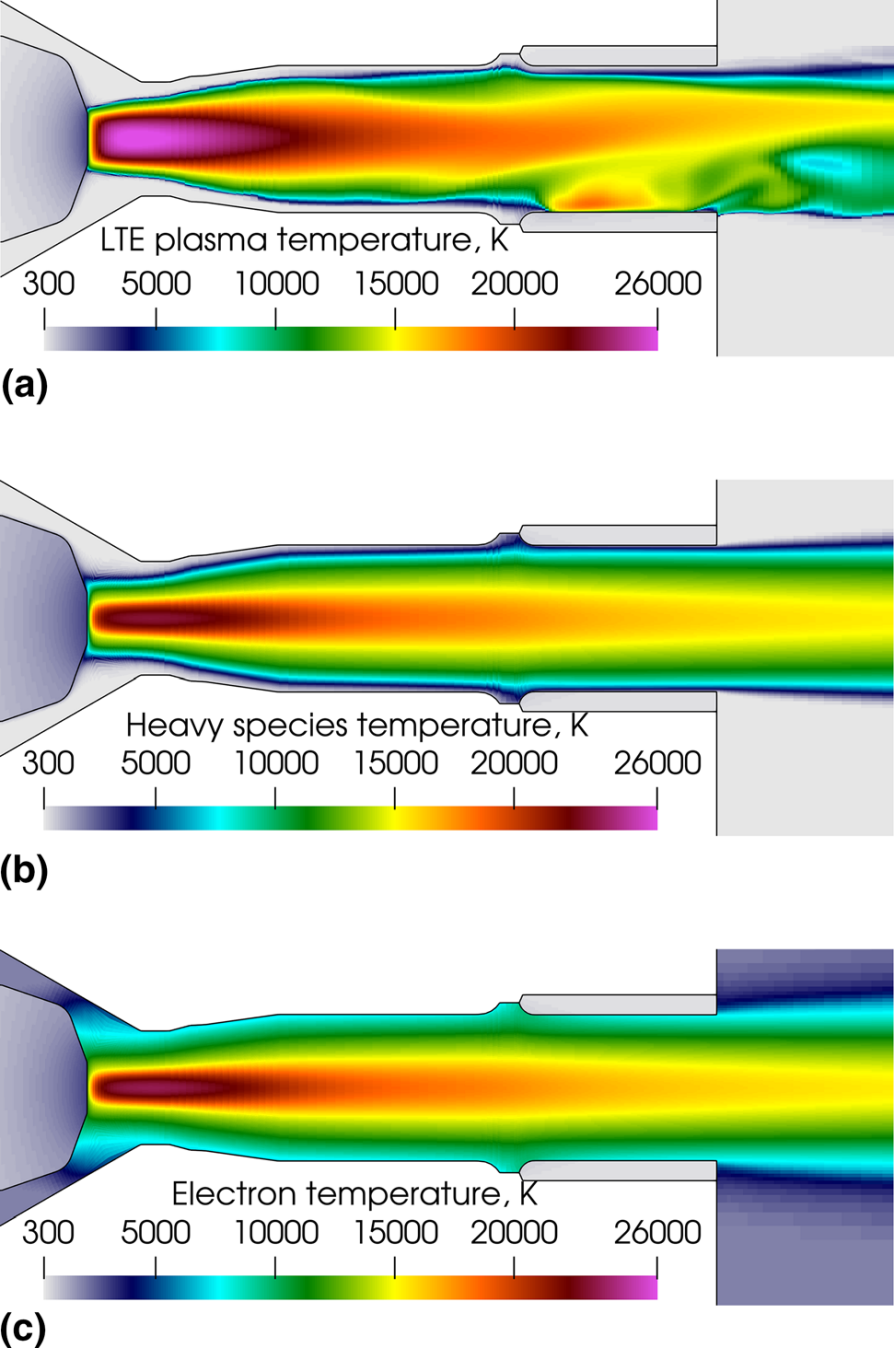
Instantaneous gas temperature fields in a vertical plane along the torch axis (5.20 ms after the ignition of the arc). (a) LTE plasma temperature, (b) heavy species temperature (Th) and (c) electron temperature (Te) predicted by the 2-T model. 500A, 60 NLPM of argon

Figure 15 shows the instantaneous plasma temperature fields predicted by the LTE (Fig. 15a) and 2-T models (Fig. 15b: heavy species temperature and Fig. 15c: electron temperature).

The temperature field of the plasma in the LTE model and heavy species in the 2-T model reflects the electric current density fields (Fig. 3a, b). Actually, the plasma is essentially heated by the resistive energy dissipation by the arc current flowing through the plasma. The LTE temperature field is clearly three-dimensional while the 2-T field is axisymmetric. In both models, the highest temperatures were close to the cathode where the current density was the highest; however, they were lower in the 2-T model yielding a lower rate of radiation heat transfer to the cathode (0.2 kW against 0.4 kW for the LTE model). A zone of high temperature was also observed close to the anode arc attachment in the LTE model.

**Fig. 16:**
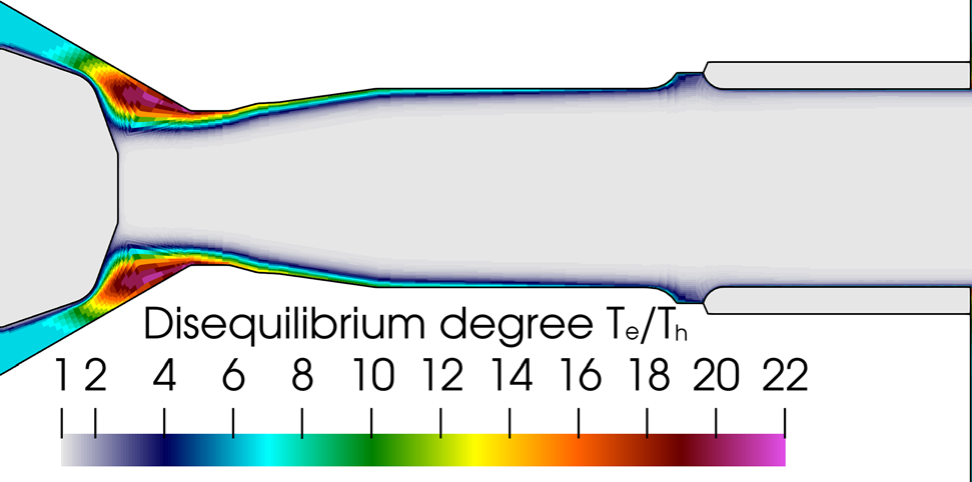
Disequilibrium degree (ratio of electron temperature to heavy species temperature) distribution in the 2-T model. 500A, 60 NLPM of argon

The comparison of Fig. 15(a) and (b) shows the cold gas boundary layer at the neutrode and anode walls while the electrons had a temperature of about 8000 K in the fringes of the arc, which is high enough to ensure a current flow between the arc column and anode. The disequilibrium between the electron and heavy species was the highest in the area around the cathode tip where the cold plasma-forming gas interacted with the hot cathodic plasma jet as seen in Fig. 16 that shows the ratio of electron temperature to heavy species temperature (disequilibrium degree θ). The same observation was made by Trelles et al. for a conventional plasma torch (Ref 31). It could be partly explained by the insufficient energy exchange between the electrons and heavy species when the electron temperature was below 8000 K (Ref 62).

**Fig. 17:**
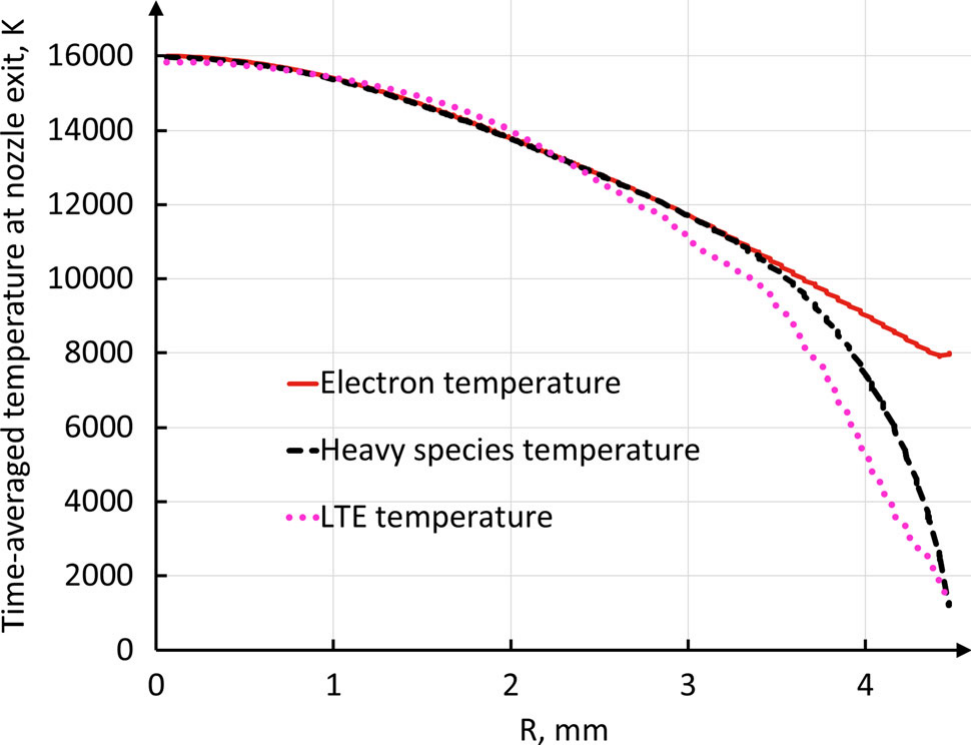
Time-averaged temperature profile at nozzle exit in the LTE and 2-T models

Figure 17 shows the time-averaged profiles of the plasma temperature at the nozzle exit. It should be reminded that in this work, depending on the model, the anode arc attachment exhibited a different mode (constricted attachment in the LTE model and diffuse in the 2-T model) and a different time-behavior (moving axially and circumferentially in the LTE model and steady in the 2-T model). The 2-T model predicted an axisymmetric distribution at the nozzle exit at each instant, while the LTE prediction became axisymmetric after averaging over time. Therefore, the comparison of the LTE and 2-T profiles was not obvious. However, contrarily to the observations drawn from the comparison of LTE and 2-T models for a conventional plasma torch (Ref 31), the exit profiles of the plasma temperature predicted by the LTE model and that of the heavy species predicted by the 2-T model were very similar. A possible explanation was the neutral insert between the cathode and anode that forced the arc to extend until the anode ring where it attached and, thus, brought about a hot and long plasma jet. In both models, the plasma temperature was over 12000 K in about 40% of the exit plane of the torch and reached 16000 K on the torch axis. This compares (Ref 66) to a maximum temperature of 10746 K calculated by Trelles et al. at the exit of a SG 100 plasma torch operated with pure argon (60 slpm) at 800 A (Ref 31) and of 13031 K calculated by Guo et al. at the exit of a F4 plasma torch operated with a mixture of argon (40 slm) and hydrogen (8 slm) at 400 A (Ref 67).

**Fig. 18:**
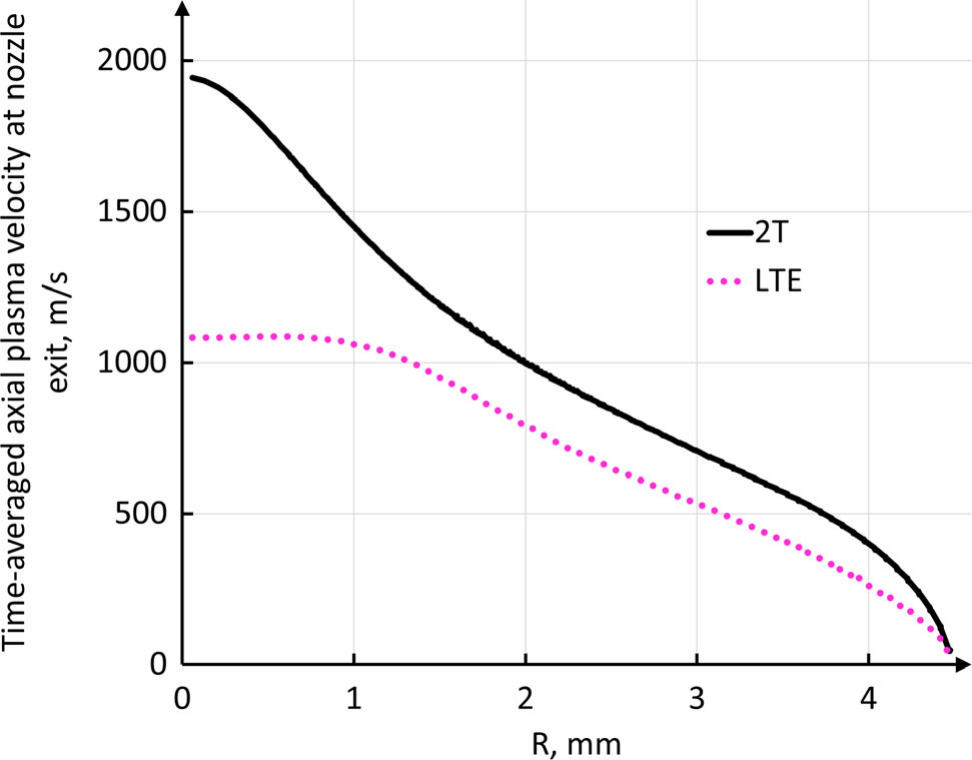
Time-averaged plasma velocity profile at nozzle exit in the LTE and 2-T models.

Figure 18 shows the time-averaged radial profiles of plasma velocity at the nozzle exit calculated by the LTE and 2-T models. They present marked differences in both the curve shape and maximum values. In the 2-T model, the plasma velocity on the torch axis reached 1950 m/s while it was 1050 m/s in the LTE model. In addition, the LTE time-averaged profile exhibited a flatter profile than the 2-T profile while the instantaneous LTE profiles presented peaks up to 1600 m/s at different points of the nozzle exit depending on the location where the arc attached.

## Conclusion

This study aimed to compare the anode arc attachment modes in a DC non-transferred plasma torch predicted by an LTE and two-temperature arc models. The three-dimensional and time-dependent models included the electrodes in the computational domain and calculated the electromagnetic and temperature fields both in the electrodes and fluid phase. The models were applied to a commercial cascaded-anode plasma torch and used the exact inner design and materials of the electrodes. The torch was operated with an arc current of 500 A and a gas flow rate of 60 NLPM of argon. Argon was selected as plasma-forming gas as the 2-T model requires the thermodynamic and transport properties of the plasma for a large panel of disequilibrium degree (ratio between the electron temperature and heavy species temperature).

Under the torch operating conditions used in this work, the LTE model predicted a constricted arc attachment that moved on the anode wall because of the swirling injection of the plasma-forming gas. This arc mode was promoted by the model used to ensure the current flow through the anode cold gas boundary layer, whose temperatures are too low to have a high enough electrical conductivity. The 2-T model did not require an additional model at the anode wall as the thermal non-equilibrium due to insufficient collisions between electron and heavy species made the cold boundary layer close to the anode significantly more electrically conductive and predicted a steady and diffuse arc attachment on the upstream edge of the anode. The post-mortem observation of a new anode operated under the same conditions for 5 min seemed to confirm the predictions of the 2-T model. Also, the predicted 2-T arc voltage (including the anode and cathode voltage drops) was 75 V, much closer to the measured arc voltage (about 71 V) than the LTE arc voltage (93 V).

The 2-T model yielded more realistic results than the LTE model and will be used for future developments. A remaining question is the anode arc attachment mode for gas mixtures with diatomic gases that are generally used for ceramics coating deposition (Ref 68). However, the current limiting step is the calculation of the non-equilibrium thermodynamic and transport properties of the gas mixtures for a large panel of diatomic gas content and disequilibrium degree. Further improvement of the 2-T model also includes the incorporation of a cathode sheath model, which should improve the prediction of the cathode arc attachment.
